# Unpacking Number Word Comprehension in Early Childhood

**DOI:** 10.1162/OPMI.a.376

**Published:** 2026-09-20

**Authors:** Alex M. Silver, Leanne Elliott, Heather J. Bachman, Elizabeth Votruba-Drzal, Melissa E. Libertus

**Affiliations:** Department of Psychology, Learning Research and Development Center, University of Pittsburgh, Pittsburgh, PA, USA; American Institutes for Research, Arlington, VA, USA; Department of Health and Human Development, School of Education, University of Pittsburgh, Pittsburgh, PA, USA

**Keywords:** number word acquisition, Approximate Number System, Object Tracking System, early childhood, math development

## Abstract

Children recognize number words prior to understanding the exact numbers, but the limits and mechanisms of this comprehension are unclear. One prominent account suggests that the Approximate Number System (ANS) provides the basis for the number word acquisition process such that symbolic number words are mapped onto imprecise numerical representations, whereas an alternative account suggests that the Object Tracking System (OTS) forms the foundation for the process as children first learn to map number words to exact, small quantities in the subitizing range. Recent theoretical proposals and evidence from longitudinal studies suggest that both the ANS and OTS may be involved in the process, however, how the distinct representations from the ANS and OTS are coordinated in real-time remains untested. Here, children (*N* = 143, *M*_age_ = 3.73 yrs) mapped number words to the referred quantities in a two-alternative forced-choice task where trials were designed with varying ratios and sizes of response options. In line with theories that the innate representational systems for both large, imprecise quantities (i.e., the Approximate Number System) and small, exact sets (i.e., the Object Tracking System) support number word acquisition, comprehension was ratio- and size-dependent, consistent with signatures of the two innate numerical representational systems. These findings provide evidence of the proposed real-time coordination of the ANS and the OTS during number word comprehension.

## INTRODUCTION

Two-and-a-half-year-old Emma sits at the table and counts the number of crackers her father has given her for her snack: “One, two, three.” Her father asks: “How many crackers do you have?” When Emma responds, “one, two, three”, her father asks: “Can you give me two crackers?” and Emma hands him all three. In this scenario, although able to correctly recite the count list, Emma does not yet understand that the last number in the count list refers to the total number of items in the set and that each number word refers only to a numerosity of that exact amount.

Scenes such as this are common occurrences in early childhood. Understanding number words is one of the earliest learned symbolic math skills (Libertus et al., 2020). Number word comprehension involves mapping the word label for each number to a representation of the number it denotes. Mature number knowledge is defined by understanding the cardinality principle—that each number word refers only to an exact set of that quantity with the last number in the count list referring to the total number of items in the set (Carey, 2009; Wynn, 1990). Children typically do not fully grasp the cardinality principle until three or four years of age (Wynn, 1990, 1992). Just like little Emma, although children may have some preliminary knowledge of how numbers are used, they are not able to produce sets of a specified numerosity for many months after learning to recite the number words. Prior to understanding the cardinal principle (CP)—and becoming a “CP-knower”—children progress through a protracted process where they slowly learn the first few number words one at a time (Wynn, 1990, 1992). During this intermediate phase, where they understand some but not all number words, children are referred to as “subset-knowers” (Le Corre & Carey, 2007). While all children acquire the number words in the same order (first learning “one,” then “two,” and then “three,” and so on) the rate of this learning differs (Geary et al., 2019).

Importantly, understanding number words is crucial for later symbolic math skills (see Geary & van Marle, 2018; Geary et al., 2018; Sarnecka & Carey, 2008). Earlier acquisition of number words is associated with better performance in other foundational math skills, including counting, number comparison, number symbol identification, arithmetic, and understanding of number lines (Geary & van Marle, 2018). Even years later, children who had more advanced understanding of number words tend to perform better in symbolic math than their peers with less advanced number knowledge (Geary et al., 2018).

### Number Word Acquisition May Not Be Discontinuous

Wynn’s (1990, 1992) proposal of number word acquisition, based on performance in the Give-N task where children are asked to produce sets of objects differing in number, posited a stepwise development. Specifically, children first learn the number “one” as a “one-knower” who can create sets of one but no larger. After several months children then become a “two-knower” who can create sets of one or two but no larger. This is followed by becoming a “three-knower” several additional months later, who can create sets of one, two, or three, but no larger. Sometime after becoming a “three-knower,” children transition to being a “four-knower” and then eventually a CP-knower.

Recent work indicates that the process of number word acquisition may be more complex than previously suggested (e.g., Gunderson et al., 2015; Huang et al., 2010; Krajcsi & Fintor, 2023; O’Rear et al., 2020; Posid & Cordes, 2018; Silver et al., 2021; Wagner et al., 2019). These studies suggest that instead of adhering to a strictly stage-like development, number word acquisition may be a more fluid and dynamic process. Specifically, children seem to have a preliminary understanding of number words prior to gaining a fully reliable and exact representation of the numerosity associated with a given number word. For example, studies examining performance in the Give-N task find that children show partial knowledge of the next number above their defined knower-level that is not yet fully consistent (O’Rear et al., 2020), but this preliminary understanding of number words seems to be limited only to small numbers (Wagner et al., 2019). Using a two-alternative forced choice task containing numbers 1–10, another study found that performance above knower-level was overall above chance when children were asked to point to a set with a specific number of objects (Silver et al., 2021). However, when examining this pattern based on children’s knower-level in Give-N, it was found that performance on numbers above the knower-level was only above chance for subset-knowers of two and higher (one-knowers did not differ from chance). These results suggest children may be able to approximately identify numbers and/or rule out seemingly very incorrect responses (e.g., the number word “three” cannot denote a set of ten objects). However, it remains unclear how far this approximate understanding of number words extends (i.e., whether subset-knowers are able to rule out that “eight” cannot refer to a set of twenty objects) and how those number words become mapped exactly onto specific quantities.

### What Representational System(s) Support Number Word Acquisition?

Extensive discussion has centered around how children learn to map number words to their referred quantities (see Carey & Barner, 2019; Sella et al., 2021 for reviews). Researchers have posited several prominent theoretical accounts for the learning of number words, and specifically the process for transitioning from a subset-knower who understands only one or a few number words to a CP-knower who understands the cardinal principle for all numbers within their count list. These accounts differ not only in the amount of learning that is required (i.e., how much of the number knowledge is innate), but also the underlying representational system that is responsible for supporting the learning process.

Under one account, researchers have proposed that the basis of all natural numbers is innate, such that number concepts are not constructed through development (e.g., Butterworth, 2005; Gelman & Butterworth, 2005; Gelman & Gallistel, 1986; Leslie et al., 2008). Instead, it is proposed that the process of learning the number list allows for expressing the number concepts that are already present. However, most researchers take slightly more constructivist views, focusing instead on how number concepts are generated from non-symbolic representations. By these accounts, even before children acquire counting and other early math skills, they have access to two distinct systems of numerical representation: the Approximate Number System (ANS) and the Object Tracking System (OTS). It is widely theorized that either one of these systems, or perhaps both, may support the number word acquisition process; however, which system is responsible is highly debated.

Some accounts propose that the Approximate Number System (ANS) provides the foundation for this process, such that symbolic number words are mapped onto the imprecise numerical representations of the ANS (e.g., Dehaene, 2009; Gallistel & Gelman, 1992, 2000; Mussolin et al., 2016; Nieder, 2017; Piazza, 2010; Sasanguie et al., 2013; Starr et al., 2013; Verguts & Fias, 2004; Wagner & Johnson, 2011). The ANS supports the ability to estimate large quantities non-symbolically (Dehaene et al., 1998; Libertus & Brannon, 2009). Present across phylogeny and throughout ontogeny, the ANS uses imprecise, noisy mental representations to produce estimates of set sizes. Discrimination of sets using the ANS is ratio-dependent, such that performance is more accurate for sets with a larger ratio than for sets with a smaller ratio between them. For example, an individual’s ability to discriminate 8 from 16 dots would be equivalent to their discrimination of 10 from 20 dots, while their discrimination of 8 from 12 dots would be more challenging and less accurate. Precision of the ANS typically improves throughout childhood, such that discrimination becomes more accurate for smaller ratios as children age (Halberda & Feigenson, 2008). Additionally, individual differences in ANS acuity exist, such that individuals vary in the precision of their ANS representations starting in infancy (Libertus & Brannon, 2010). These individual differences in the ANS are related to variability in symbolic math skills from early childhood through adulthood (Chen & Li, 2014; Fazio et al., 2014; Halberda et al., 2012; Schneider et al., 2017; Starr et al., 2013). It is argued that with age, as the ANS becomes more precise, the mapping between number words and the underlying non-symbolic representations in the ANS becomes more refined to allow for the accurate mapping of the number words to their referred quantities.

Meanwhile, others propose that the Object Tracking System (OTS) plays the critical role as children learn to map number words to small quantities within the subitizing range first, and only over time do they learn to extend this process to larger numbers (e.g., Barner, 2012, 2017; Carey, 2004; Carey & Barner, 2019; Carey et al., 2017; Gunderson et al., 2015; Le Corre & Carey, 2007; Sarnecka, 2021). The Object Tracking System (OTS) supports the ability to individuate small numbers of objects (Feigenson et al., 2004). Present from birth, the OTS allows for temporary storage of information about the perceptual properties of objects in “object files”. The OTS allows for one-to-one correspondence between objects and object files in memory, which allows for exact representation and tracking of small quantities (Feigenson & Carey, 2003). However, the OTS is constrained with memory limits, such that individuals are able to exactly discriminate and represent up to only three or four objects without the use of other memory strategies (Feigenson & Carey, 2005). Like the ANS, the OTS is generally thought to be a universal system that is present across ontogeny and phylogeny (see Feigenson et al., 2004). Importantly, infants are unable to discriminate quantities represented by the ANS and OTS respectively (e.g., 4 and 2) suggesting that these systems are distinct and do not support cross-system comparisons (Lipton & Spelke, 2004; but see Cordes & Brannon, 2009). Under the OTS account, the parallel individuation of objects made possible by the OTS allows children to learn the cardinality principle by first mapping small numbers to their quantities. Children must then learn the logic of how counting sets allows for mapping numbers to quantities. This logic requires children to apply the correct order of the number words when counting, map each number word to one and only one object (i.e., one-to-one correspondence), and understand that adding one object to a set will result in a set that is labeled with the next number word in the sequence. Over time and only once they become CP-knowers, children eventually learn to associate large number words in the count list with approximate magnitudes.

Finally, it is possible that both the ANS and OTS may be involved in the process of number word acquisition and cardinal principle learning (e.g., Feigenson et al., 2004; Spelke, 2011; van Marle et al., 2018). Specifically, the OTS may produce exact representations for small numbers, while the ANS supports cardinal values for a larger range of magnitudes. These representations may then be combined (potentially via language) to support verbal counting and learning of the cardinal principle and other counting principles (see Condry & Spelke, 2008). van Marle et al. (2018) provided the first longitudinal evidence supporting this view, showing that while both systems seem to be related to cardinal knowledge early on, only the ANS predicted the acquisition of the cardinal principle, and after this milestone the ANS was the only system related to cardinal knowledge. In that study, individual differences in performance on tasks measuring the ANS and OTS were related to variability in later cardinal number knowledge, but OTS tasks did not predict cardinal knowledge after acquisition of the cardinal principle. These results suggest that both systems may contribute to children’s early number word comprehension, but the OTS becomes less relevant over time. However, how the distinct representations from the ANS and OTS are coordinated in real-time (especially prior to acquisition of the cardinal principle) remains unknown. It may be that the ANS is recruited for representation of some numbers (e.g., large numbers), while the OTS is recruited for others (e.g., small numbers). Some evidence for such a dissociation exists—children who know only a few number words but have not yet mastered the cardinal principle seem unable to extend their knowledge of number to larger cardinal values (Condry & Spelke, 2008), suggesting the two systems may work independently. On the other hand, it remains possible that both the ANS and OTS may be involved in representing all numbers, or it may be that which representational system is recruited differs depending on some other features of the task or stimuli.

Moreover, in addition to varying in theoretical explanations, previous studies have primarily relied on children’s ability to produce sets of objects as an indicator of their number knowledge, but this approach may underestimate children’s competence (see Cordes & Gelman, 2005; Frye et al., 1989; O’Rear et al., 2020; Silver et al., 2021; Wagner et al., 2019; but see Le Corre et al., 2006). Here, we examine a less frequently used pointing task to test what affects children’s performance and shed light on the underlying mechanism for number word comprehension. The process of how children become a CP-knower is only one piece of the puzzle, and the process of number word comprehension for a broader set of numbers warrants deeper attention (see Krajcsi et al., 2023). In particular, given previous findings that both the ANS and OTS are related to early number word comprehension, it is critical to understand how these systems coordinate to support numerical representations. However, this question has been often overlooked in favor of debate about the process for acquiring the cardinal principle. Here we aim to compare number word comprehension for a broad range of numbers rather than predicting CP-knower status, in an attempt to disentangle the extent to which there is continuity/discontinuity in how children comprehend and produce number words and how the ANS and OTS may uniquely or jointly support number word comprehension. In many other cases of language development, if a child can produce a word, they also comprehend it (and indeed, comprehension tends to come much earlier than production; Goldin-Meadow et al., 1976). In the case of number words, that is not the case. Children can often recite number words much earlier than they comprehend the meaning of those words (see Libertus et al., 2020).

### The Current Study

The current study considers how young children like little Emma in the introductory example, who have not yet acquired cardinal-principle knowledge but can recite some number words in the correct order, map number words to the referred quantities in a two-alternative forced choice Point-to-X task (e.g., Silver et al., 2021; Wynn, 1992). Children were shown two images containing sets of identical stimuli differing only in number (e.g., four ducks and five ducks) and were asked to point to one of the images containing a specific number of objects (e.g., “Which has four ducks?”). We replicate and extend previous work using the Point-to-X task with a larger set of numbers and additional ways of incorporating children’s performance on other number knowledge tasks. While previous work has indicated that children show preliminary understanding of numbers above their Give-N knower-level, the limit for this understanding has not yet been thoroughly explored (as previous work was conducted using a fairly truncated range of number options which did not allow for testing comprehension of numbers far beyond the current knower-level; O’Rear et al., 2020; Silver et al., 2021). Specifically, using two indices of children’s number knowledge, we take a more comprehensive look at whether number word comprehension extends above children’s Give-N knower-level and mastery of the count list to identify whether comprehension extends broadly to many numbers above children’s current knowledge or is limited to only the next few numbers. To this end, we analyze performance in trials containing numbers that are within their knowledge range, trials containing numbers above their knowledge range, and trials containing numbers slightly above their knowledge range. In this set of preliminary analyses, we ask:

Prelim RQ1: Does Point-to-X performance differ as a function of children’s knower-level? Do children display partial knowledge of numbers above their Give-N knower-level in the Point-to-X task?
Prelim RQ2: Does Point-to-X performance differ as a function of children’s mastery of the count list? Do children display partial knowledge of numbers above their count list in the Point-to-X task?

To answer the primary research questions, in addition to examining overall performance, Point-to-X trials were designed with varying ratios and sizes of the response options to test whether performance was ratio-dependent (in line with predictions of ANS support) or size-dependent (in line with predictions of OTS support). If performance in Point-to-X is significantly predicted by the ratio of the response options, such that performance is better for larger ratios than smaller ratios (a hallmark of ANS magnitude comparison), this would support the ANS account. Meanwhile, if performance is significantly predicted by the size of the response options, such that performance is better for smaller numbers than larger numbers (those within the tracking range of the OTS versus outside the OTS range), this would support the OTS account. We also test how the strength of these effects may differ based on the size and ratio of numbers or children’s age, as well as children’s cardinal number knowledge (as indexed by Give-N performance) and mastery of the count list. Specifically, in primary analyses we consider:

RQ1: Is performance in the Point-to-X task modulated by both the ratio and size of the response options? Is the ratio- and size-modulation in the Point-to-X task consistent across trials of various number sizes and ratios?
RQ2: Does the strength of the ratio and/or size modulation differ as a function of children’s age?
RQ3: Does the ratio- and size-modulation in the Point-to-X task hold when controlling for children’s knower-level as measured via the Give-N task, and does the strength of the ratio and/or size modulation differ as a function of children’s knower-level?
RQ4: Does the ratio- and size-modulation hold when controlling for children’s highest count as measured via a verbal counting task, and does the strength of the ratio and/or size modulation differ as a function of children’s highest count?

## METHODS

### Participants

Participants were 143 children (46% males) ranging in age from 3 years, 4 months to 4 years, 3 months (*M* = 3 years, 9 months, *SD* = 3 months). Descriptive statistics reporting sample demographic information are displayed in Table 1. Parents reported their family income, where 21% of families were classified as low-income (i.e., earnings below 200% of the poverty line), 34% as middle-income (i.e., earning between 200 and 399% of the poverty line), and 45% as high-income (i.e., earnings 400% and above of the poverty line). All families spoke English.

**Table 1.** Descriptive statistics for sample demographic information, *N* = 146.

**Variable**	***M* (*SD*)**	**Min, Max**
Child age in years	3.73 (0.23)	3.34, 4.24
Family income in $10,000s	10.32 (5.88)	0.20, 30
**Variable**	***N***	**% of sample**
Child gender		
Female	77	54%
Male	65	46%
Not reported	1	1%
Child race/ethnicity		
Asian	4	3%
Black/African American	14	10%
Hispanic/Latino	5	4%
Other/Multiracial	6	4%
White, Non-Hispanic	111	78%
Not reported	3	2%
Child’s mother’s education		
Did not finish high school	1	1%
High school diploma or vocational/technical degree	11	8%
Some college but no degree	15	10%
Associate’s degree	6	4%
Bachelor’s degree	45	31%
Graduate degree	65	46%
Child’s father’s education		
Did not finish high school	1	1%
High school diploma or vocational/technical degree	27	19%
Some college but no degree	6	4%
Associate’s degree	11	8%
Bachelor’s degree	52	37%
Graduate degree	43	31%
Not reported	3	2%

An additional 24 children participated but were dropped from analyses due to failure to answer the two practice items in the Point-to-X task correctly (*n* = 8), failure to complete the full Point-to-X task (*n* = 6), or failure to complete the full Give-N task (*n* = 10). Families were recruited from Pittsburgh, Pennsylvania, a midsized city in the Northeastern United States, through a research participant registry and a combination of flyers, online postings, and in-person recruitment at childcare centers or community events from September 2021 to November 2023. Prior to any data collection parents provided written informed consent as approved by the local Institutional Review Board.

### Procedure

Data were drawn from the Parents Promoting Early Learning Study, a longitudinal study investigating the role that parents play in promoting young children’s acquisition of early math skills. Data for the present study were collected during the second wave of the longitudinal study via a Zoom video call where children completed a Point-to-X task, a Give-N task, and a verbal counting task (see details below). The assessments were video recorded and coded by trained researchers. In addition, children also completed assessments measuring their spatial skills, vocabulary, executive functioning, and number skills (e.g., recognition of number symbols), and parents also completed brief assessments of their math and reading fluency, as well as an online survey about demographic information, home activities, beliefs about early learning, and other characteristics of parents and families. These measures were not related to the current study aims and are thus not further described here.

### Materials

#### Point-to-X.

A Point-to-X task similar to the one used by Silver et al. (2021) was used to measure children’s comprehension of number words. For each trial, children viewed two images side by side (see Figure 1 for example stimuli and Table 2 for full list of items). Each image had a small label in the top corner with a Roman numeral I or II, and parents were instructed to indicate which choice the child pointed to by holding up one or two fingers or by verbally labelling the child’s choice.

**Figure 1.**
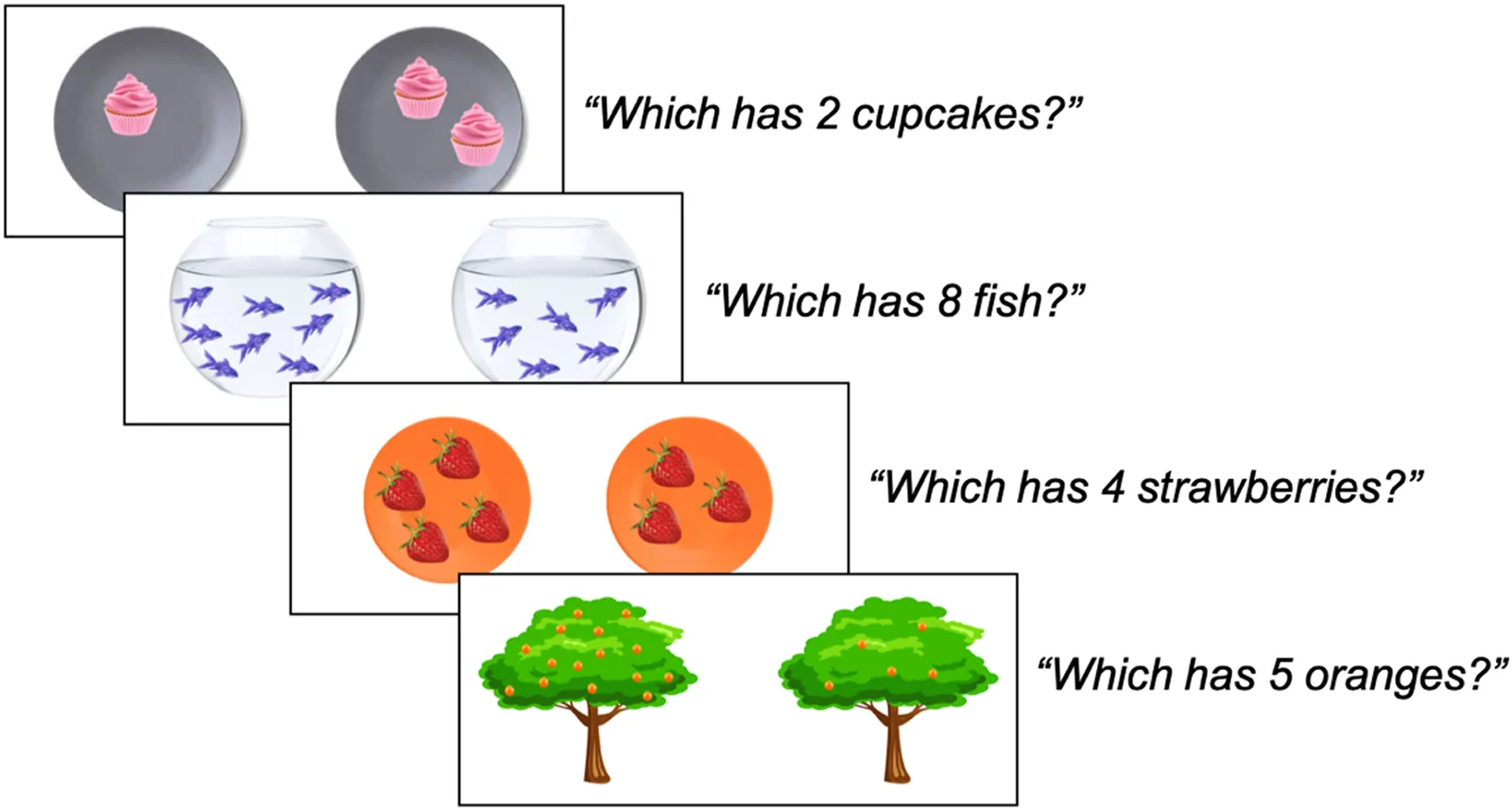
Example stimuli from the Point-to-X task, including a trial with both small response options in a large ratio (1 vs. 2 cupcakes), a trial with both large response options in a small ratio (6 vs. 8 fish), a trial with both small response options in a small ratio (3 vs. 4 strawberries), and a trial with both large response options in a large ratio (5 vs. 15 oranges).

**Table 2.** Trial prompts for Point-to-X task.

**Trial Number**	**Prompt**	**Display Left**	**Display Right**	**Ratio of Options**	**Size of Options**	**Size of Target**
P1	Which has a ball?	Car	ball	–	–	–
P2	Which has a tree?	Tree	cup	–	–	–
1	Which has 2 cupcakes?	1	2	Large	Both Small	Small
2	Which has 8 fish?	8	6	Small	Both Large	Large
3	Which has 16 ants?	16	20	Small	Both Large	Large
4	Which has 1 duck?	3	1	Large	Both Small	Small
5	Which has 15 bees?	15	12	Small	Both Large	Large
6	Which has 10 birds?	5	10	Large	Both Large	Large
7	Which has 9 peppers?	9	12	Small	Both Large	Large
8	Which has 4 ladybugs?	5	4	Small	One Small	Small
9	Which has 3 frogs?	3	6	Large	One Small	Small
10	Which has 12 cherries?	4	12	Large	One Small	Large
11	Which has 8 cookies?	10	8	Small	Both Large	Large
12	Which has 4 fish?	4	2	Large	Both Small	Small
13	Which has 16 flowers?	12	16	Small	Both Large	Large
14	Which has 2 pigs?	2	6	Large	One Small	Small
15	Which has 4 strawberries?	4	3	Small	Both Small	Small
16	Which has 5 oranges?	15	5	Large	Both Large	Large

To familiarize children with the task, children were given two practice trials with two different common objects and prompted to point to one image (e.g., “Which has a ball?”). Subsequently, in sixteen number-word trials, each trial showed two sets of identical stimuli differing only in number (e.g., four ducks and five ducks), and children were prompted to point to one of the images (e.g., “Which has four ducks?”). Number-word trials varied along three dimensions: (1) the numerical ratio between the two sets (for four trials each the numbers differed by a 1:3, 1:2, 3:4, and 4:5 ratio); (2) the size of the target number (for seven trials the prompted number was small [1–4], and for nine trials the prompted number was large [5–20]); and (3) the size of the response options (for four trials both numbers were small numbers [1–4], for four trials one number was small [1–4] and one was large [5–20], and for eight trials both numbers were large numbers [5–20]). The side of the screen of the correct response and the size of the target in the pair (whether it is the smaller or larger number presented) were counterbalanced across trials.

When administering the task, if children initially pointed to one image, then verbally indicated that they wanted to change their answer, the second point was counted as their response. In cases where children did not respond, the experimenter repeated the prompt up to two times and said, “Just give your best guess.” If children still did not respond, the experimenter moved on to the next trial and children received 0 points for the trial. If children pointed to both images without clearly signalling their response, the experimenter prompted, “Just give your best guess. Which has [number] [noun]?” After this prompt, if children continued to point to both images, they received 0 points for the trial.

Responses were coded by trained research assistants to identify which image children pointed to for each trial (based on the response that parents signalled). Children received 1 point for selecting the correct image, or 0 points for choosing the incorrect image. 20% of videos were double-coded by a second researcher to assess inter-coder reliability (overall scores between coders were highly correlated, *r* = .95, *p* < .0001). Children’s Point-to-X score is the percentage of trials for which they correctly pointed to the requested number.

#### Give-N.

A Give-N task similar to the one used by Silver et al. (2021) and based on Wynn’s seminal work (Wynn, 1990, 1992) was used to classify children’s “knower-level” score. Families were sent a set of the materials (a plate and 10 plastic toy fish) prior to the testing session. During the Zoom call children were shown a bear puppet held up by the experimenter and a large pile of plastic fish. They were asked to help “feed” the bear by putting out the correct number of fish for the puppet to eat on the plate. The researcher asked children to “feed” the bear different numbers of fish by placing the fish in a pile. For each trial, children were asked “Can you give the bear [number] fish?” and instructed to put the set of fish on the plate for the bear to eat. After the child paused for more than 3 s or indicated that they were done creating the set, the experimenter prompted confirmation from the children, “Is that [number] fish?” If children said yes or nodded, the experimenter held the bear up to the webcam and said, “Yum yum yum! Thank you!” If children said no or shook their head, they were given one chance to correct their response and were instructed, “Ok, well the bear wants [number] fish. Can you give the bear [number] fish?” Once children had adjusted the number of fish or paused for more than 3 s, the experimenter held the bear up to the webcam and said “Yum yum yum! Thank you!” Children’s parents helped facilitate the clearing of the plate after each trial by returning the fish to the main pile before the next trial. If children did not respond to a trial, the experimenter repeated the prompt one time. If children still did not respond, the experimenter moved on to the next trial and children were considered to have responded incorrectly and received zero points for that trial.

Trials were administered in a titrated manner (see Wynn, 1990, 1992). All children were first asked for one fish and then for two fish. If a child correctly responded to a trial, they were then tested with the next number in the sequence (e.g., asked for three after responding correctly to two). If a child responded incorrectly to a trial, they were subsequently asked for the next smaller number (e.g., asked for one after responding incorrectly to two). This process was repeated until children successfully produced a set of *N* fish twice and failed to produce *N* + 1 twice. If children failed to give even one fish correctly, the task ended after they failed when asked for one twice and they were given a score of 0. If children successfully gave six fish, they were not asked for more but instead asked for six fish again to test their reliability. If they succeeded twice at producing six fish, the task ended, and they were given a score of 6. Children were not given any feedback on their performance, and the highest number at which they produced the correct set size twice while failing twice at the next highest number was used here as their Give-N “knower-level” score (in line with Marchand et al., 2022). 20% of videos were double-coded by a second researcher to assess inter-coder reliability. Scores between coders were perfectly correlated, *r* = 1.00, *p* < .0001.

#### Verbal Counting.

Children were asked to count as high as they could, specifically “Let’s count! Can you count for me?”. If children did not respond, the experimenter prompted, “Count like this with me: one…, two…, three…, Can you keep going? Count as high as you can.” If children remained silent, the experimenter added, “Can you count your fingers?” If children successfully counted all of their own fingers, the experimenter held up their fingers so children could count their fingers as well. Children were then offered the option of counting their parents’ fingers as well. If children stopped their correct counting, the experimenter prompted, “What comes next?” The task was ended when children made their first error or they stopped counting and maintained that they could not count any higher. The task ended for all children with the experimenter saying “Great! Thanks for counting!” The highest number children successfully counted to was recorded. 20% of videos were double-coded by a second researcher to assess inter-coder reliability (overall scores between coders were highly correlated, *r* = .99, *p* < .0001).

### Planned Analyses

All analyses were conducted using Stata/SE 15.1 (StataCorp, 2017) and RStudio (Posit Team, 2023). As this was a new format of administration with new trials for the Point-to-X task compared to previous studies, we first examined descriptive statistics for children’s overall performance in the Point-to-X task.

#### Preliminary Analyses.

To first test whether children’s performance in the task was significantly above chance, we used a *t*-test comparing the mean performance across all trials to 50% (i.e., expected performance if children were guessing for each trial). We then conducted a series of preliminary analyses based on children’s Give-N knower-level and highest count to determine if performance was above chance for trials within their knower-level or count list (i.e., containing at least one number that was smaller or equal to their knower-level or highest count) and above their knower-level or count list (i.e., containing only numbers larger than their knower-level or highest count). Moreover, given the large range of numbers tested on the Point-to-X task, we broke this down to examine trials that contained numbers larger but just above children’s knower-level or highest count (i.e., knower-level + 2 or highest count + 2) and numbers much larger than children’s knower-level or highest count (i.e., knower-level + 3 or more or highest count + 3 or more) to test if children had some comprehension of numbers above their knower-level or count list and if it did or did not extend to much larger numbers.

#### Primary Analyses.

Next, to address our main research questions, we examined children’s performance on trials of various ratios and with response options of different sizes to determine whether performance was ratio-dependent (as might be predicted by ANS accounts) or size-dependent (as might be predicted by OTS accounts). We tested whether performance was significantly modulated by the ratio or the size of response options (RQ1), and whether this ratio and/or size modulation was moderated by children’s age (RQ2), knower-level (RQ3) or highest count (RQ4) by adding interactions between each of these three variables and both the ratio and the size of the response options, respectively (more details about each model are presented below).

Using mixed effects modeling, we tested and compared a series of models using the *lme4* package in R (Bates et al., 2007). Prior to analyses we standardized continuous variables, and all dichotomous variables were coded using effects coding to allow for ease of interpretation of results. For all models, random effects included intercepts for each individual child (*n* = 143) and each trial (*n* = 16) to account for clustering within children and within trials. The maximal models were initially tested for all mixed effect models. In cases where the models failed to converge, to maintain the maximal random effects structure, the correlation parameters were removed from the models. In cases where the models converged but they remained overfitted as indicated by a “singular fit” warning, to further reduce model complexity, the random slopes were removed. Model comparison indicated that models not containing random slopes or correlations better fit the data as indicated by lower Akaike Information Criterion (AIC) and Bayesian Information Criterion (BIC). Therefore, for parsimony, all final models did not include the random slopes or correlations.

First, we predicted performance in a given trial from fixed effects of the size of the response options (either both small or both large), the ratio between response options (either small or large ratio), and children’s age, with random effects for individual children and individual trials (RQ1). We then added an interaction term between the size of the response options and the ratio between response options to see whether associations differ for the different types of trials. Next, we added two interaction terms (one between children’s age and size of the response options, and one between children’s age and ratio of the response options) to see whether associations differ based on children’s age (RQ2). Then, we added main effects of children’s knower-level (RQ3) or highest count (RQ4) to the baseline model to see if the effect of ratio and/or size held when controlling for children’s number knowledge. Finally, we added interaction terms between children’s knower-level (RQ3) or highest count (RQ4) and size and ratio of the response options to see whether associations differ based on children’s knower-level or mastery of the count list.

## RESULTS

### Preliminary Analyses: Overall Performance in the Point-to-X Task

Descriptive statistics are presented in Table 3. As expected, given previous results (Silver et al., 2021), we found that overall performance in Point-to-X was significantly better than chance, *M* = 70.50 (*SD* = 15.41), *t*(142) = 15.91, *p* < .0001. 86.01% of the sample (123 children) scored above chance (> 50% correct) overall. This is particularly striking given the larger range of numbers used in the current version of Point-to-X compared to previous versions of the task.

**Table 3.** Descriptive statistics for number tasks.

	**1**	**2**	**3**
**1 Point-to-X (*N* = 143)**	–		
**2 Give-N Knower-Level (*N* = 143)**	.53***	–	
**3 Highest Count (*N* = 129)**	.35***	.34***	–
***M* (*SD*)**	70.50 (15.41)	4.063 (1.86)	14.40 (12.63)
**Min, Max**	31.25, 100	0, 6	0, 101

*** *p* < .001.

#### Preliminary RQ1: Performance in the Point-to-X Task as a Function of Give-N Knower-Level.

We first identified children’s knower-level in Give-N (Figure 2a) and found that Point-to-X performance was significantly correlated with knower-level, *r* = .53, *p* < .0001 (Figure 3). Performance in the Point-to-X task as a function of children’s knower-level is displayed in Table 4. Performance was significantly above chance for trials containing at least one number within children’s knower-level (i.e., for a 1-knower, trials that included “one” as an option; for a 2-knower, trials that included either “one” or “two”), *M* = 87.92 (*SD* = 17.88), *t*(140) = 25.18, *p* < .0001. We then looked at performance for trials including numbers above children’s knower-level (e.g., for a 1-knower, all trials where the smallest number was any number larger than “one”; for a 2-knower, all trials where the smallest number was larger than “two”). Performance on trials including numbers above their knower-level was significantly above chance, *M* = 53.30 (*SD* = 19.03), *t*(142) = 2.07, *p* = .040, and a one-way ANOVA suggested performance on trials containing numbers above the knower-level did not differ based on knower-level, *F*(6, 1452) = 0.66, *p* = .684. It is possible that although children scored above chance for numbers above their knower-level, this is driven by preliminary knowledge of the next one or two numbers (see O’Rear et al., 2020) rather than a preliminary understanding of all larger numbers. As such, we looked at performance for trials including numbers one or two above children’s knower-level (e.g., for a 1-knower, trials where the smallest number was “two” or “three”; for a 2-knower, trials where the smallest number was “three” or “four”) and trials including numbers far above children’s knower-level (e.g., more than two above the knower-level). Performance on trials containing numbers one or two above children’s knower-level was significantly above chance, *M* = 56.87 (*SD* = 39.13), *t*(142) = 2.10, *p* = .037, and a one-way ANOVA suggested performance on trials containing numbers one or two above the knower-level did not differ based on children’s knower-level, *F*(6, 142) = 0.81, *p* = .564. Performance on trials containing numbers greater than two above children’s knower-level was not significantly different from chance, *M* = 50.88 (*SD* = 22.04), *t*(142) = 0.48, *p* = .634, and a one-way ANOVA suggested performance on trials containing numbers far above the knower-level did not differ based on children’s knower-level, *F*(6, 142) = 1.50, *p* = .181.

**Figure 2.**
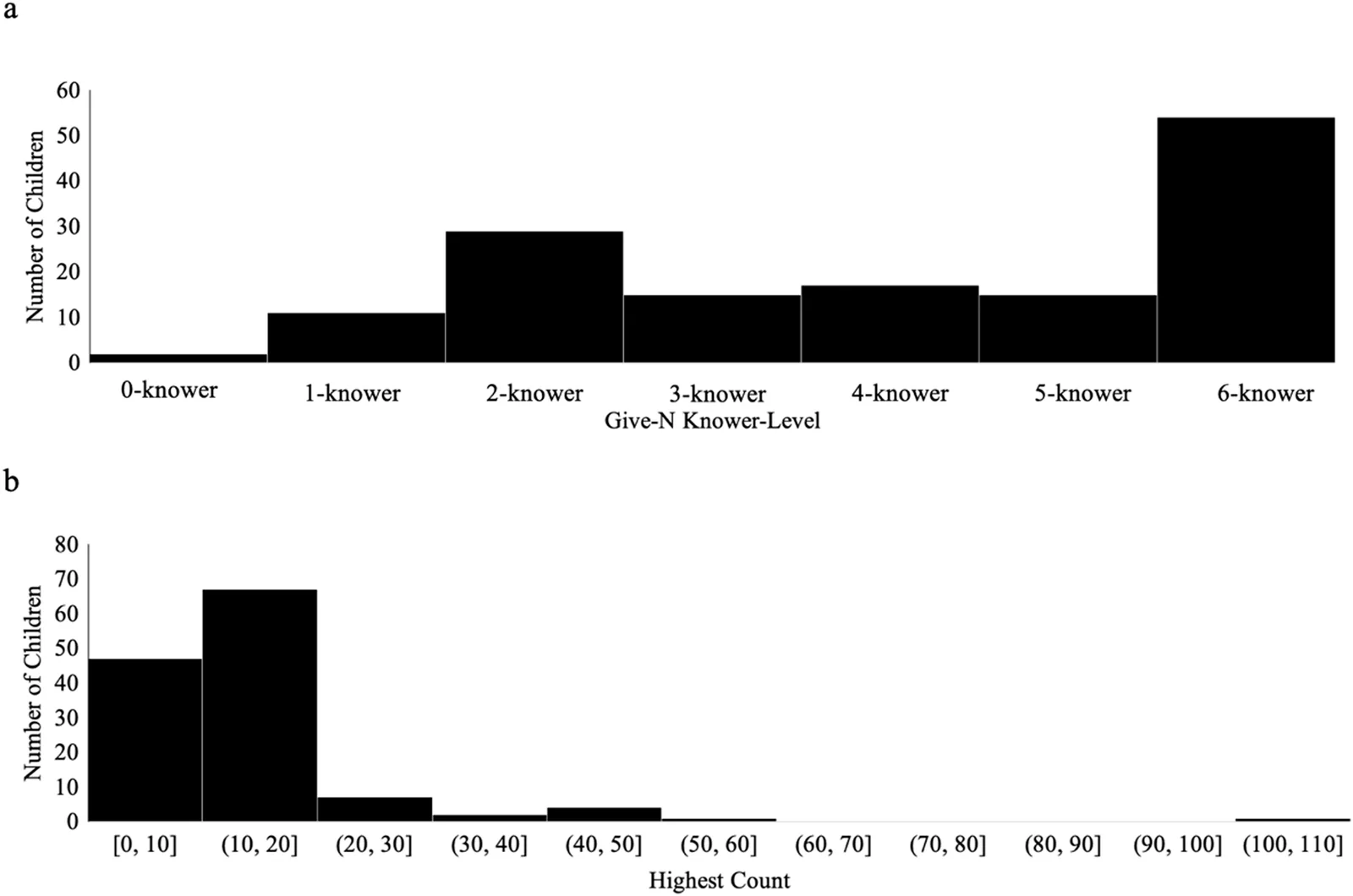
Histograms of children’s Give-N knower level (a) and highest count (b).

**Figure 3.**
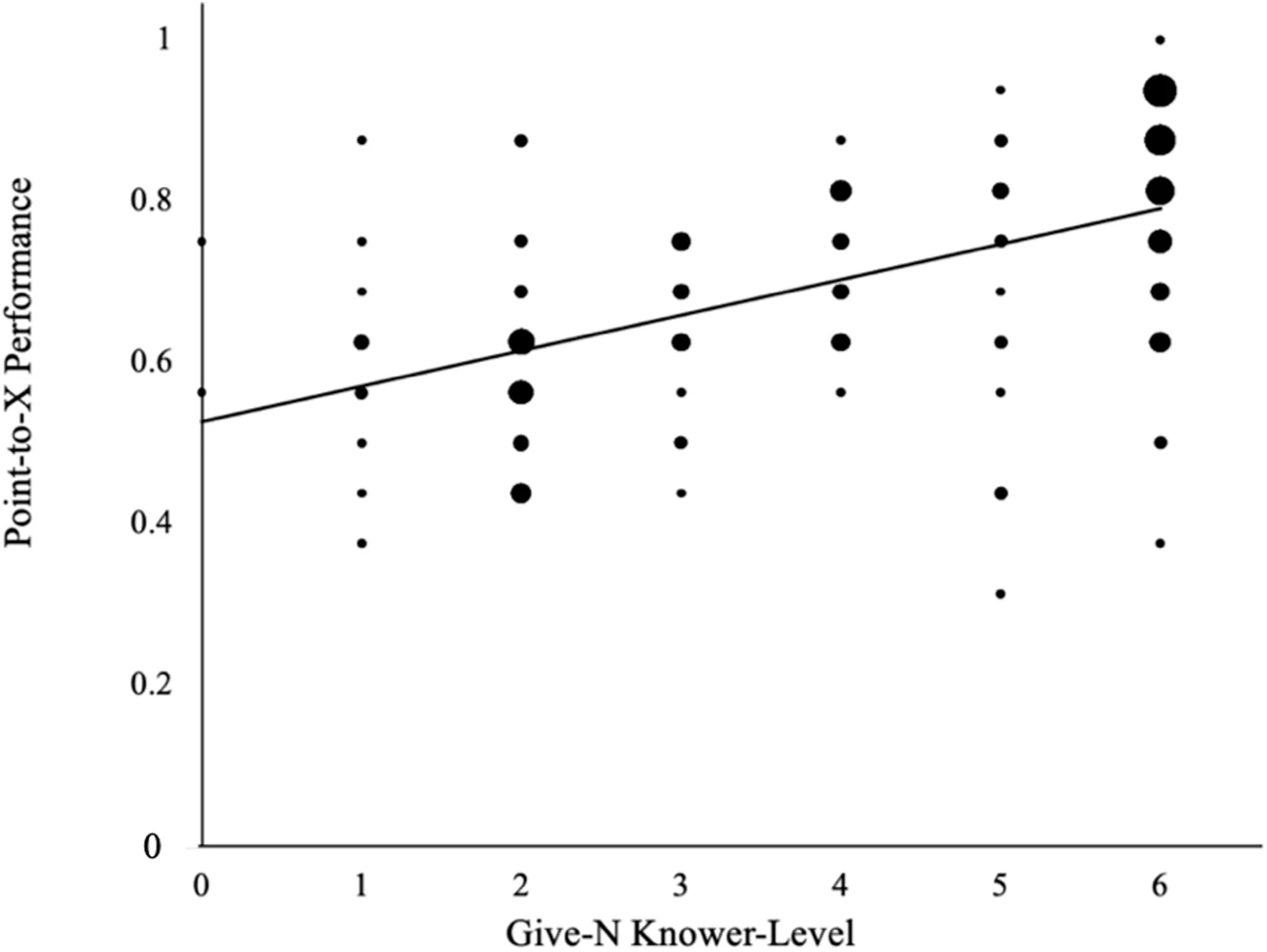
Point-to-X performance as a function of children’s Give-N knower level.

**Table 4.** Point-to-X performance as a function of children’s knower-level (*N* = 143).

**Knower-Level**	**Point-to-X Overall Performance**	**Point-to-X Trials Within Knower-Level**		**Point-to-X Trials Above Knower-Level**		**Point-to-X Trials One or Two Above Knower-Level**	
	***M* (*SD*)**	***N* Trial**	***M* (*SD*)**	***N* Trial**	***M* (*SD*)**	***N* Trial**	***M* (*SD*)**
0-knower (*n* = 2)	65.63 (13.26)	0	–	16	65.63 (13.26)	4	87.50 (17.68)
1-knower (*n* = 11)	60.23 (14.05)*	2	95.45 (15.08)****	14	55.19 (15.01)	4	54.55 (26.97)
2-knower (*n* = 29)	59.48 (11.77)***	4	79.31 (24.15)****	12	52.87 (12.85)	4	64.66 (25.46)**
3-knower (*n* = 15)	63.75 (10.08)***	6	86.67 (20.12)****	10	50.00 (9.26)	4	63.33 (18.58)*
4-knower (*n* = 17)	72.43 (9.13)****	8	91.91 (8.77)****	8	52.94 (13.64)	3	60.78 (21.20)^†^
5-knower (*n* = 15)	68.75 (18.45)***	10	82.00 (21.11)****	6	46.67 (21.08)	1	46.67 (51.64)
6-knower (*n* = 54)	80.44 (13.25)****	11	91.75 (12.77)****	5	55.56 (24.78)	1	51.85 (50.43)

*Note*. Significant difference from chance indicated by asterisks.

^†^ *p* < .10,

* *p* < .05,

** *p* < .01,

*** *p* < .001,

**** *p* < .0001.

#### Preliminary RQ2: Performance in the Point-to-X Task as a Function of Highest Count.

Of the 143 children included in analyses, 129 completed the verbal counting task (Figure 2b). Thirty children were able to count to the highest number queried in the Point-to-X task (i.e., 16), while the remaining 99 children could not. One child counted to 100 or more, which was considered an outlier (> 3 *SD* above the mean), and thus was excluded from the following analyses using highest count. Of the 128 children included in highest count analyses, children’s highest count ranged from 0 to 59. Children who could count to all numbers queried (*M* = 78.45, *SD* = 15.81) performed significantly better than those who could not count to all numbers (*M* = 68.18, *SD* = 14.54) in the Point-to-X task, *F*(1, 127) = 10.75, *p* = .001, Cohen’s *d* = 0.68, although performance for both groups was significantly above chance, *t*(28) = 9.69, *p* < .0001, and *t*(98) = 12.44, *p* < .0001, respectively. Point-to-X performance was significantly correlated with children’s highest count, *r* = .37, *p* < .0001. Performance in the Point-to-X task as a function of children’s highest count is displayed in Table 5.

**Table 5.** Point-to-X performance as a function of children’s highest count (*N* = 128).

	***M* (*SD*)**
**Highest Count**	13.73 (10.06)
**Point-to-X Trials Within Count List**	75.07 (16.63)****
**Point-to-X Trials Above Count List**	42.98 (37.63)^†^
**Point-to-X Trials One or Two Above Count List**	56.22 (43.01)

*Note*. Significant difference from chance indicated by asterisks.

^†^ *p* < .10,

**** *p* < .0001.

Performance was significantly above chance for trials containing at least one number within children’s count list (i.e., for a child who could count to 1, trials that included “one”; for a child who could count to 2, trials that included “one” or “two”), *M* = 75.07, *SD* = 16.63, *t*(125) = 16.92, *p* < .0001. A one-way ANOVA suggested that performance on Point-to-X trials that included at least one number within children’s count list did not differ based on the highest number children could count to, *F*(28, 125) = 1.23, *p* = .227. We then looked at performance for trials including numbers above children’s highest count (i.e., for a child who could count to 1, all trials where the smallest number was any number larger than “one”; for a child who could count to 2, all trials where the smallest number was larger than “two”). Performance on trials including numbers above children’s count list was not significantly different from chance, *M* = 42.98, *SD* = 37.63, *t(*98) = −1.86, *p* = .066, and a one-way ANOVA suggested this did not differ based on children’s highest count, *F*(15, 98) = 0.91, *p* = .552. Finally, we examined performance for trials including numbers one or two above children’s highest count (i.e., for a child who could count to 1, all trials where the smallest number was “two” or “three”; for a child who could count to 2, all trials where the smallest number was “three” or “four”). Performance on trials containing numbers one or two above children’s count list was not significantly different from chance, *M* = 56.22, *SD* = 43.01, *t*(62) = 1.15, *p* = .256, and a one-way ANOVA suggested performance did not differ based on children’s highest count, *F*(12, 62) = 1.40, *p* = .200.

### Primary Analyses

#### RQ1: Testing for Ratio-Dependent and Size-Dependent Performance.

Next, we explored whether children’s performance was modulated by the ratio between response options or the size of the response options (see Figure 4). In all models we included random effects of individual children and trials, which together accounted for 41.3% of the variance in performance on a given trial (6.8% of the variance was due to variability across children, and 34.5% of the variance was due to variability across trials).

**Figure 4.**
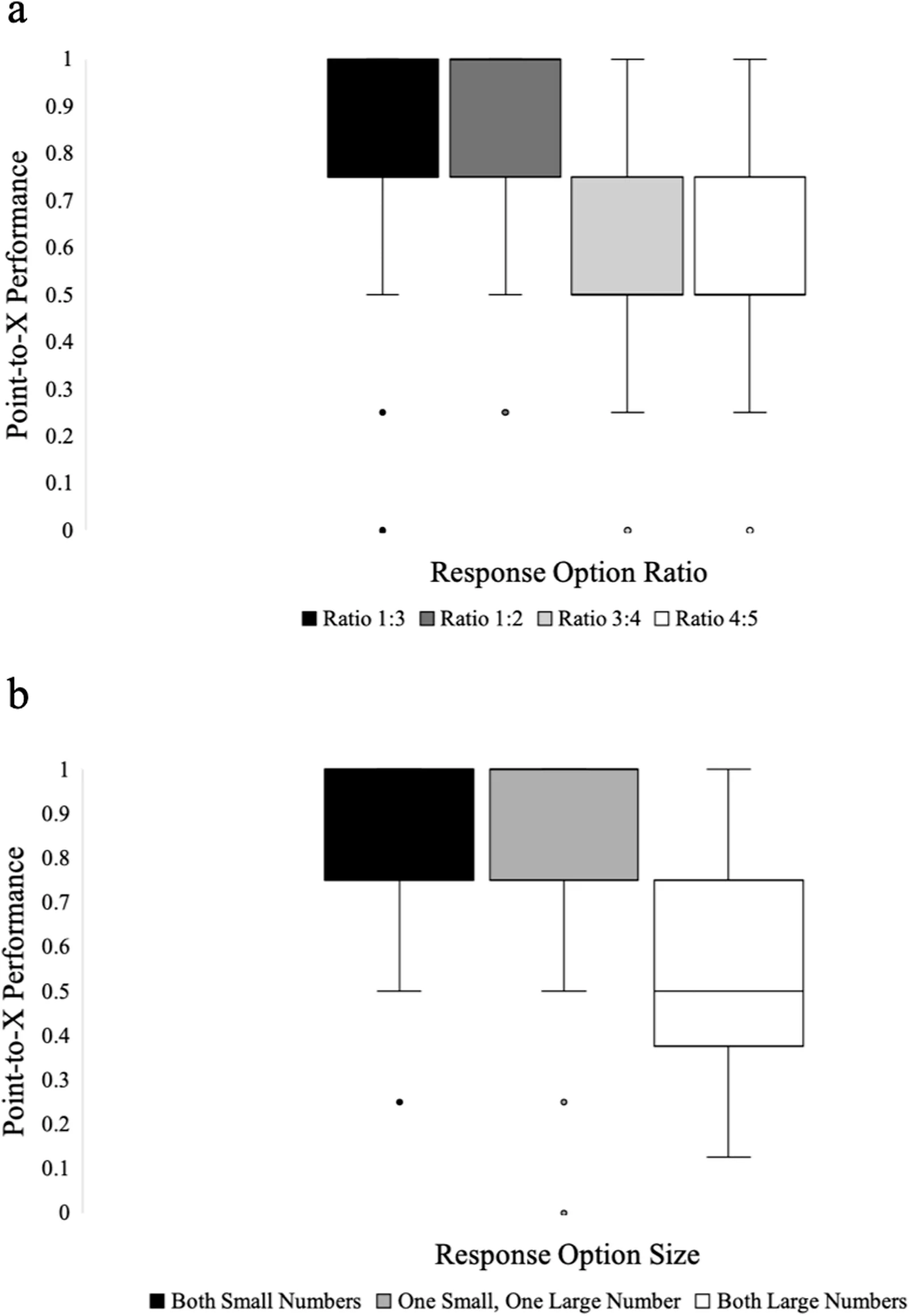
Point-to-X performance on trials of different ratios (a) and sizes (b).

First, we predicted performance in a given trial from fixed effects of the size of the response options, ratio of the response options, and child’s age. We found significant effects of size (such that trials where both options were small increased the odds of being correct by 5.16 times), ratio (such that trials where the ratio between response options was large increased the odds of being correct by 3.63 times), and child’s age (such that older children performed better than younger children). Given the findings in our Preliminary Analyses suggesting that performance may differ based on whether a trial contained numbers within children’s knower-level/count list, we included additional predictors to control for these individual differences. Results remained consistent when including additional predictors for whether trial response options fell within children’s knower-level and count list, as well as when including predictors for whether trial response options fell one or two above children’s knower-level and count list (such that there remained significant effects of both size and ratio on performance).

Next, we added an interaction term between the size and ratio of response options. The pattern of significant effects from the original model remained, and no significant interaction was seen between the size and ratio of response options. Results from these models can be found in Table 6.

**Table 6.** Results from generalized linear mixed effects models predicting performance in a given Point-to-X trial.

**Fixed effect**	**Model 1**	**Model 2**	**Model 3**
	***B* (*SE*)**	***B* (*SE*)**	***B* (*SE*)**
Intercept	1.44 (0.23)****	1.41 (0.25)****	1.53 (0.23)****
Child age	0.29 (0.08)***	0.29 (0.08)***	0.49 (0.10)****
Response option ratio	−1.27 (0.46)**	−1.33 (0.49)**	−1.34 (0.47)**
Response option ratio × Child age	–	–	−0.27 (0.15)^†^
Response option size	−1.65 (0.49)***	−1.63 (0.49)***	−1.78 (0.50)***
Response option size × Child age	–	–	−0.35 (0.18)^†^
Response option ratio × Response option size	–	0.29 (0.98)	–
**Random effect**	***SD***	***SD***	***SD***
Child intercept	0.54	0.57	0.54
Trial intercept	0.66	0.64	0.66

*Note*. All estimates are in log odds.

^†^ *p* < .10,

* *p* < .05,

** *p* < .01,

*** *p* < .001,

**** *p* < .0001.

#### RQ2: Testing the Strength of Ratio- and Size-Dependence as a Function of Children’s Age.

We then tested whether the strength of the ratio- and size-dependence differed based on children’s age. To the baseline model of main effects (which included ratio of the response options, size of the response options, and child’s age), we added interaction terms between children’s age with the ratio of response options and the size of response options. The pattern of significant effects from the baseline model remained, and there were no significant interactions between children’s age and the ratio and size of response options. Results from this model can be found in Table 6.

#### RQ3: Testing the Strength of Ratio- and Size-Dependence as a Function of Knower-Level.

We next turned to testing whether the strength of the ratio- and size-dependence differed based on children’s knower-level. We first examined the main effects, where we found significant effects of the ratio of response options, the size of response options, children’s age, and children’s knower-level (such that performance was better for children with higher knower-levels). We added interaction terms between the ratio and size of response options with knower-level to determine if the associations differed based on children’s knower-level. We found that all significant effects from the original model of main effects remained, and a significant interaction emerged between knower-level and the size of the response options, such that larger effects of knower-level were seen for trials where both numbers were small than for trials where both numbers were large. Specifically, the effect of knower-level increased the odds of being correct by 7.61 times more for trials where both response options were small compared to trials where both response options were large. No such interaction was found between knower-level and the ratio of response options. Results from these models can be found in Table 7.

**Table 7.** Results from generalized linear mixed effects models predicting performance in a given Point-to-X trial.

**Fixed effect**	**Model 1**	**Model 2**	**Model 3**	**Model 4**
	***B* (*SE*)**	***B* (*SE*)**	***B* (*SE*)**	***B* (*SE*)**
Intercept	1.44 (0.22)****	1.68 (0.24)****	1.47 (0.22)****	1.63 (0.24)****
Child age	0.18 (0.07)*	0.18 (0.08)*	0.25 (0.08)**	0.24 (0.08)**
Knower-level	0.36 (0.07)****	0.70 (0.11)****	–	–
Highest count	–	–	0.36 (0.10)***	0.81 (0.19)****
Response option ratio	−1.28 (0.46)**	−1.36 (0.48)**	−1.27 (0.45)**	−1.39 (0.46)**
Response option ratio × Knower-level	–	−0.13 (0.15)	–	–
Response option ratio × Highest count	–	–	–	−0.45 (0.26)^†^
Response option size	−1.65 (0.49)***	−2.08 (0.53)****	−1.69 (0.48)***	−1.93 (0.50)***
Response option size × Knower-level	–	−0.86 (0.19) ****	–	–
Response option size × Highest count	–	–	–	−0.72 (0.36)*
**Random effect**	***SD***	***SD***	***SD***	***SD***
Child intercept	0.42	0.46	0.48	0.49
Trial intercept	0.65	0.68	0.63	0.64

*Note*. All estimates are in log odds.

^†^ *p* < .10,

* *p* < .05,

** *p* < .01,

*** *p* < .001,

**** *p* < .0001.

#### RQ4: Testing the Strength of Ratio- and Size-Dependence as a Function of Highest Count.

Finally, we repeated this same series of analyses using children’s highest count. We found the same general pattern of results as with knower-level (with main effects of ratio, size, age, and highest count in the model of main effects, significant interaction of highest count with the size of response options, and no interaction between highest count and the ratio of the response options). The effect of highest count increased the odds of being correct by 6.55 times more for trials where both response options were small compared to trials where both response options were large. Results from these models can be found in Table 7.

### Robustness Checks

In addition to the analyses described in the main text, we considered additional analyses which are presented in the Supplemental Material. We tested whether ratio- and size-dependent effects differed based on whether trials including the number 1 were excluded to examine whether singular/plural markings contributed an additional cue to quantity and contributed to children’s success. This was not the case (see Robustness Analysis 1). We also tested whether ratio- and size-dependent effects differed based on whether the number 4 was classified as a small or large number. This was also not the case (see Robustness Analysis 2).

## DISCUSSION

Over time young children like Emma in the introductory example, who can recite numbers when asked to count but cannot yet create sets of requested sizes, eventually learn to recite the count list in order, recognize number words, and map number words to quantities. Understanding how children learn to map number words to their referred quantities has led to extensive debate in the field (see Carey & Barner, 2019; Sella et al., 2021). With implications for variability in math achievement later in life (e.g., Duncan et al., 2007; Jordan et al., 2009), and subsequent consequences for education, career, financial and health outcomes (e.g., Agarwal & Mazumder, 2013; Currie & Thomas, 1999; Reyna & Brainerd, 2007), exploring individual differences in the earliest symbolic math skills is necessary. Moreover, understanding the mechanism for acquiring early symbolic math skills such as acquisition of number words is crucial to identifying the best ways to support children’s learning of these skills. Previous work comparing theories for how children acquire number words have typically focused on explanations for how children come to understand the cardinality principle. Here, we focus on how children comprehend number words broadly, rather than on this critical step of cardinal principle understanding. Given that more complex number knowledge (such as understanding cardinality) builds on this foundational skill, understanding the process of mapping the first numbers to their referred quantities is critical for supporting children’s early mathematical skills.

### Partial Knowledge of Number Words Before Gaining Exact Understanding

In line with other recent work (e.g., Gunderson et al., 2015; Huang et al., 2010; O’Rear et al., 2020; Posid & Cordes, 2018; Silver et al., 2021; Wagner et al., 2019), our findings in the preliminary analyses suggest that young children may have some understanding of number words prior to mastering their exact meanings. Specifically, children perform above chance at identifying the correct quantity amongst two choices when given a specific number word. As in previous work (e.g., Silver et al., 2021), we find that even if they cannot yet create sets of the numerosities for all number words queried, children nonetheless may have some preliminary understanding that allows them to rule out incorrect options or approximately map the number word to the referred quantity more often than would be expected if they were simply guessing. This is particularly meaningful considering that the range of numbers presented in the current version of the Point-to-X task ranged from 1-20, whereas previous studies included numbers only up to 10 at most (Silver et al., 2021; van Marle et al., 2014). Yet, children remained successful at identifying the correct quantity when given a specific number word in the current Point-to-X task, suggesting they may have some preliminary knowledge of the number words. Indeed, children may be able to use this preliminary knowledge of number words to extend the range of numbers they can contrast through mutual exclusivity (see Condry & Spelke, 2008).

Previous work has suggested that Give-N is a very stringent test of number word comprehension and although children may not be able to accurately produce the exact number of objects when asked, they do possess preliminary understanding of number words above their knower-level (e.g., O’Rear et al., 2020; Silver et al., 2021; Wagner et al., 2019). However, there may be differences in the processes used to identify number words above knower-level based on the size of the number or children’s current number knowledge. Although children scored above chance overall on trials containing numbers above their knower-level, we found that this was primarily driven by their above-chance performance on trials containing numbers one or two above their knower-level. Performance on trials containing numbers farther above knower-level did not differ from chance. Critically, this suggests a limit to children’s preliminary understanding of numbers. Rather than extending infinitely, the approximate understanding of number words seems limited to only numbers slightly above children’s knower-level (but still in their count list). These results may suggest that the Give-N task underestimates children’s true number knowledge (as they seem to comprehend numbers slightly above their knower-level). Although previous work has suggested this approximate understanding of numbers is limited to small numbers (e.g., Wagner et al., 2019), we find that it extends at least up to two numbers above the knower-level even for three-knowers and possibly for four-knowers (although it should be noted that there was only one possible trial for 5- and 6-knowers that probed numbers one or two above their knower-level so it remains unclear whether with additional trials we would see similar patterns for those children as well). This suggests that children may learn the (approximate) meaning of number words denoting numerosities beyond four prior to learning the cardinal principle (see Carey et al., 2017; Cheung et al., 2016; Condry & Spelke, 2008; Gunderson et al., 2015; Le Corre & Carey, 2007; Odic et al., 2015; Wagner & Johnson, 2011; Wagner et al., 2019, for other studies of children’s understanding of large number words prior to learning the cardinal principle with mixed results). These results seem to support the proposed distinction in conceptual processes between comprehension of number words on the one hand and learning to count and understand counting procedures on the other hand (e.g., Cheung et al., 2025; O’Rear et al., 2020; Wagner et al., 2019).

Meanwhile, in contrast to the knower-level analyses, children’s performance on trials above their count list was not significantly different from chance even for numbers only slightly above their count list. Although children were able to succeed on Point-to-X trials containing numbers up to two above their knower-level, children performed no different from chance on trials containing numbers one or two above their count list. Mastery of the count list was ultimately a fairly weak indicator of children’s number word comprehension. Altogether, these results suggest that Give-N may be more conservative in indicating children’s number word comprehension as children seem to have some partial comprehension of numbers above their knower-level, while mastery of the count list may be a more liberal (and perhaps too liberal) indicator of children’s number word comprehension. As each of these measures of children’s number skills is associated with different strengths and drawbacks, these findings reinforce the importance of utilizing multiple measures to assess children’s developing number knowledge.

### What Underlying Cognitive Mechanism(s) Support Number Word Comprehension?

How this preliminary knowledge becomes exact and the underlying representational system that supports this process is still an open question. Researchers have argued that the underlying mechanism for number word acquisition may be based in the approximate representations of quantity of the ANS (e.g., Dehaene, 2009; Gallistel & Gelman, 1992, 2000; Mussolin et al., 2016; Nieder, 2017; Piazza, 2010; Sasanguie et al., 2013; Starr et al., 2013; Verguts & Fias, 2004; Wagner & Johnson, 2011), or the parallel individuation of the OTS (e.g., Barner, 2012, 2017; Carey, 2004; Carey & Barner, 2019; Carey et al., 2017; Gunderson et al., 2015; Le Corre & Carey, 2007; Sarnecka, 2021). In addition, it has been suggested that both innate numerical representation systems may be involved (Spelke, 2011, 2017; van Marle et al., 2018). Here we find that children’s number word comprehension performance is modulated by both the ratio and size of the numbers, in line with the predictions of a theory that involves both the ANS and the OTS.

Children’s earliest comprehension of number words may be supported by both numerical representational systems, as children display both ratio- and size-dependent numerical comparison performance, hallmarks of the ANS and OTS, respectively. Even when accounting for effects of the other and controlling for children’s number knowledge (as measured by both knower-level and mastery of the count list) we see that children’s performance in the Point-to-X task is modulated by both the size and the ratio of the response options in each trial. Furthermore, we saw different patterns in the moderation of size and ratio effects based on children’s knower-level and highest count, suggesting that children’s number knowledge (measured as Give-N knower-level and mastery of the count list) does not influence their ratio-dependent discrimination and instead influences only their size-dependent discrimination. Interestingly, there were no significant interactions of ratio-dependence with children’s number knowledge (either knower-level or highest count). Performance was better for trials with larger ratios, a hallmark of the ANS, regardless of children’s exact number knowledge. Children seem to be relying on less exact representations, supporting the view that the ANS supports more approximate comprehension that is independent of exact number knowledge. It is unclear whether ratio effects on number word comprehension would extend indefinitely, but it seems unlikely that children would be able to use the ANS to map number words they have never heard. To fully test how far these ratio effects extend, we would need to use a larger range of numbers including those with which children are completely unfamiliar. Critically, there were no significant interactions of the size or ratio of response options with children’s age, suggesting that any effects are driven by children’s number knowledge.

In contrast to the null interactions between number knowledge and ratio of response options, we did find a significant interaction between number knowledge and the size of response options. There was a stronger effect of number skills for trials with small numbers, suggesting that children who have a higher knower-level or who can count higher performed better on small number trials than those with lower knower-level or less words in their count list. However, this number knowledge did not seem to make as much of a difference for large number trials, suggesting children may not be using their knowledge of exact numbers to respond to trials using large numbers.

When considering the interaction between children’s knower-level and the size of response options, we find that children with higher knower-levels seemed to particularly outperform their peers with lower knower-levels in trials with small numbers. For small numbers, this makes sense, as children who have a better exact understanding of small numbers (as shown through their ability to create sets of small numerosities in Give-N) would be expected to perform better in trials that also measure knowledge of small numbers. However, why this better exact number knowledge does not also translate to better performance in trials with larger numbers is unclear. On the one hand, it could be that exact number knowledge does not help when needing to discriminate large numbers, and children rely on some other strategy to make their response. On the other hand, it could also be that the sample of children included here did not have exact knowledge of numbers larger than six. We did not test children’s ability to create sets greater than six, so it is unknown how many children truly have exact number knowledge for even larger numbers, and whether their performance on large number trials may look different than those who cannot create sets above six.

Meanwhile, our results examining the effects of highest count on the strength of the size modulation of children’s performance also showed an interesting pattern. We found that stronger mastery of the counting list (i.e., ability to correctly count higher) was particularly helpful for comparisons of small numbers. This is puzzling as we might expect that children who can count higher would be better able to use their counting knowledge to aid in comparisons of large numbers. One could imagine that children who do not fully understand number words could still use their relative positions in the count list to figure out which of two numerosities the word label maps to (employing the “later-greater” principle; see Spelke & Tsivkin, 2001). However, that does not seem to be the case here (see also Le Corre, 2014). Alternatively, it is possible that we might not see use of the later-greater principle for large number trials because children lacked the ability to count to all of the numbers used in the task (and this made comparisons particularly difficult for large numbers to which children could not yet successfully count). Children on average could only count to a subset of the numbers queried in Point-to-X, and very few children could count to all numbers. It is possible that if we had tested more children who could count to all numbers, the effect of counting skills on performance in large number trials would be just as strong as for small number trials.

Overall, our results are in line with the recent theoretical (e.g., Spelke, 2011, 2017) and empirical (Condry & Spelke, 2008; van Marle et al., 2018) accounts suggesting that both the ANS and OTS support number word acquisition (but see Krajcsi et al., 2023). Unlike in infancy when the ANS and OTS seem to operate separately (Lipton & Spelke, 2004), Spelke (2011, 2017) theorized that the OTS and ANS are combined through language to support verbal counting, which allows children to reason about cardinality. However, it remains unclear which system may support number word comprehension after children achieve this milestone. van Marle et al. (2018) similarly suggested that both the OTS and ANS are influential in the acquisition of the cardinal principle and further found that the OTS may become less influential after this milestone. While our cross-sectional findings cannot disentangle the question of longitudinal timing, we nonetheless find results in line with both of these theories.

Our findings generate novel questions about the ways in which these systems may work together and how this coordination is actively achieved. A first important direction to understand is how the two systems are employed independently and jointly in the protracted developmental process of acquiring number words. Testing the temporal sequence of how the ANS and OTS are used as young children slowly acquire the meaning of number words will be necessary to examine how prior knowledge influences the use of one or both systems. An intriguing future direction would be to examine the developmental trajectory to determine whether the ratio- and size-modulation we see here is present at all ages and knowledge levels, or whether it changes over development as suggested by van Marle et al. (2018). Specifically, do we see stronger ratio- or size-effects at some points than at others, and if so, why? Although we saw overall better performance as children’s age increased, and marginally significant interactions between children’s age and the size and ratio of response options, it seems possible that with a wider age range we might see more definitive differences in the strength of ratio- and size-modulation.

### Limitations, Conclusions and Future Directions

A few limitations of the current work warrant consideration. First, we were limited by the number of trials we could collect from participants, as young children’s attention span and cooperation is sometimes limited. As such, there were very few trials for some of the critical trial types and we were unable to fully counterbalance each number pair asking for each of the numbers. Similarly, as the small number range is constrained, it is impossible to fully disentangle the effects of the total number of items presented from the ratios being tested in the small number range (e.g., the smallest numbers in a small ratio trial, 3v4, will always include more total items being presented to children than the large ratio trials with 1v3 or 1v2 items). Additionally, our sample, although wide-ranging in education and family income, was recruited from one geographic region in the United States. Additional work including a more representative sample is certainly warranted to ensure the generalizability of these results. Finally, this study focuses on English number words, but as previous work has detailed differences in the number word acquisition process for children learning different languages (see Silver & Libertus, 2022, for review), future work testing whether similar results are found for children learning number words in other languages is needed.

A related future direction includes exploring the extent to which general language skills are related to or needed for number word acquisition. Schröder et al. (2022) found that children’s general vocabulary at 18 months predicted their number knowledge at 2.5 years of age, suggesting a key role for language in this process. However, it is unclear whether this association is due to the language-heavy task demands of number knowledge assessments (e.g., understanding the instructions to follow the commands) or a broader relation between language and number. Future work, perhaps using looking-time tasks or other methodology that does not require or rely so heavily on language and motor planning skills (i.e., where children do not need to point to their response but simply look at it or where prompts are linguistically simpler and involve looking to the quantity matching a spoken number word, e.g., “Look at two” or “Find two”; see Ambridge & Rowland, 2013, for discussion of language acquisition methodology), could help tease apart these possibilities.

Finally, understanding the developmental trajectory involved in the ratio- and size-dependence of children’s number word comprehension will be critical. Testing children from a wide range of ages to identify whether ratio- and size-modulation of numerical comparison is present at all ages, and the relative strength of each effect, is warranted to better understand how the theorized underlying representational systems may jointly support number word learning.

In sum, using a two-alternative forced choice Point-to-X task we find that 3-year-old children may partially comprehend number words beyond their exact knowledge, and this comprehension is modulated by both ratio and size of the numbers. These findings suggest that young children’s number word comprehension is more continuous than originally posited and may be supported by both the ANS and OTS.

## Acknowledgments

We thank Juliana Kammerzell, Erica Schweitzer, Margaret Isaacson, Olivia Sidoti, Taylor Montue, Natalie Heywood, Margaret Laird, Amber Liller, Dominique Lopiccolo, Grace Connors, Serena Braun, Hateya Foxx, Annette Tull, Linsah Coulanges, Shirley Duong, Danielle Fox, Jessica Ferraro, Jorge Carvalho-Pereira, Chelsea Ptak, Tamara Podvysotska, and the research assistants in the Kids’ Thinking Lab for help with data collection and data entry. Furthermore, we especially thank the families who participated. Following acceptance, the authors agree to publicly register the data, analytic code, and materials necessary to reproduce the analyses and findings presented here on the Open Science Framework. The analyses presented here were not preregistered.

## Funding Information

This work was funded by the National Science Foundation (DRL1920545 to M.E.L., H.J.B. and E.V.D.) and a Scholar Award from the James S. McDonnell Foundation to M.E.L.

## Author Contributions

A.M.S.: Conceptualization; Data curation; Formal analysis; Investigation; Writing – original draft. L.E.: Data curation; Investigation; Writing – review & editing. H.J.B.: Funding acquisition; Supervision; Writing – review & editing. E.V.-D.: Funding acquisition; Supervision; Writing – review & editing. M.E.L.: Funding acquisition; Supervision; Writing – review & editing.

## Ethics Approval Statement

This work has been approved by the Institutional Review Board of the University of Pittsburgh under protocol 19060332.

## Data Availability Statement

Data Availability Statement. The data and analysis code for this article can be found on the Open Science Framework: https://doi.org/10.17605/OSF.IO/HGJZF. The analyses presented here were not preregistered.

## Supplementary Material


