# Supplementary material for: Unpacking Number Word Comprehension in Early Childhood
Source: Open Mind (Camb). 2026 Sep 20;10:1525–49. doi: 10.1162/OPMI.a.376 (PMC13733549; doi:10.1162/OPMI.a.376)
Supplement: Supplementary file 1 [file opmi-10-1525-s001.docx]

**Supplemental Material**

**Additional Robustness Checks**

***Robustness Analysis 1: Excluding Trials with One Object as a Response Option***

Prompts in the Point-to-X task included nouns (which could be either plural or singular, i.e., “Which has ten birds?” or “Which has one duck”?). This led any trial where “one” was a response option to include an additional linguistic cue to quantity beyond the number words, and it is possible that this redundant cue aided in children’s ability to discriminate “one” from other numbers. As such, we excluded all trials that included one object as a response option (trials 1 and 4) and examined our pattern of results for all remaining trials.

In this model, we included fixed effects of response option ratio, response option size, and child’s age with random effects for individual children and individual trials. As in our main analyses, we found significant effects of ratio (such that trials where the ratio between response options was large increased the odds of being correct by 2.55 times, *p* = .024), size (such that trials where both options were small increased the odds of being correct by 2.97 times, *p* = .024), and child’s age (such that older children performed better than younger children, *p* < .001).

***Robustness Analysis 2: Classifying “Four” as a Large Number***

Previous studies have suggested that small numbers and large numbers may be represented with different systems (e.g., Coubart et al., 2014; Mou & vanMarle, 2014; Xu, 2003), and four is often considered the boundary between small and large numbers. Work with infants suggests four objects may be represented as a large number and thus prevents accurate comparison with small numbers (e.g., Feigenson et al., 2002), whereas other studies have found that four objects can be represented and compared with small numbers (e.g., Mack, 2007), and the use of strategies like chunking allows infants to overcome the typical small number limit to include 4 (e.g., Feigenson & Halberda, 2004). Here, we originally classified “four” as a small number (see Agrillo et al., 2015), but as an extra robustness check examined whether our pattern of results holds when considering small numbers to be only those <4 and large numbers to be ≥ 4.

In this model, we again included fixed effects of response option ratio, response option size, and child’s age with random effects for individual children and individual trials. As in our main analyses, we found significant effects of ratio (such that trials where the ratio between response options was large increased the odds of being correct by 3.03 times, *p* = .013), size (such that trials where both options were small increased the odds of being correct by 12.30 times, *p* < .001), and child’s age (such that older children performed better than younger, *p* < .001).
